# Specific Ion Effects of Chaotropic and Superchaotropic Anions Probed by Raman Hydration‐Shell Spectroscopy

**DOI:** 10.1002/anie.6630432

**Published:** 2026-05-22

**Authors:** Werner M. Nau, Denilson Mendes de Oliveira, Andres S. Urbina, Harald Knorke, Jonas Warneke, Andrea Barba‐Bon, Dor Ben‐Amotz

**Affiliations:** ^1^ School of Science Constructor University Bremen Germany; ^2^ Department of Chemistry Purdue University West Lafayette, Indiana USA; ^3^ Wilhelm‐Ostwald‐Institut Für Physikalische und Theoretische Chemie Universität Leipzig Leipzig Germany; ^4^ Leibniz Institute of Surface Engineering (IOM) Leipzig Germany

**Keywords:** chaotropic effect, Hofmeister effects, hydrogen bonds, hydrophobic effect, superchaotropic ions, water structure

## Abstract

Chaotropic ions enhance protein solubility and the associated Hofmeister effects are widely used in biotechnology and materials science. However, the characterization of the recently discovered class of superchaotropic borate cluster anions remains incomplete, limiting their rational application. Using Raman multivariate curve resolution spectroscopy, we measured the O─H stretching vibrational frequencies of hydration‐shell water molecules surrounding both conventional and superchaotropic anions. For conventional monoanions, we observed dangling O—H bands in their hydration‐shell spectra, whose vibrational frequencies increase with chaotropicity and follow the trend Br^−^ < I^−^ < ClO_4_^−^, BF_4_^−^ < PF_6_^−^. This affords a useful molecular‐spectroscopic scale for ion classification and differentiation from kosmotropic and hydrophobic solutes. In contrast, superchaotropic dodecaborates (B_12_X_12_^2−^, X = H, F, Cl, Br, and I) emerged as significant outliers: their dangling O─H frequencies do not exceed those of classical chaotropes, despite displaying more pronounced chaotropic properties (such as salting‐in effects) and they show an opposing trend along the halogen series. These deviations were attributed to higher charge and charge screening in solution which affect ion‐water interactions to a different extent depending on the X substituents. These effects compete with general chaotropicity trends and affect the hydration pattern.

## Graphical abstract

The vibrational frequencies of “dangling” water O─H bonds can be determined by Raman hydration‐ shell spectroscopy and used to differentiate chaotropes from kosmotropes and hydrophobes. Dangling O─H bonds are also detected around superchaotropic anions of the borate cluster type (B_12_X_12_^2−^; X = H, F, Cl, Br, and I) but their frequencies do not directly scale with their chaotropicity order. DFT calculations show that these deviations are related to their higher net charge and charge‐screening effects.

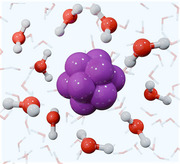

## 1 Introduction

**FIGURE 1:**
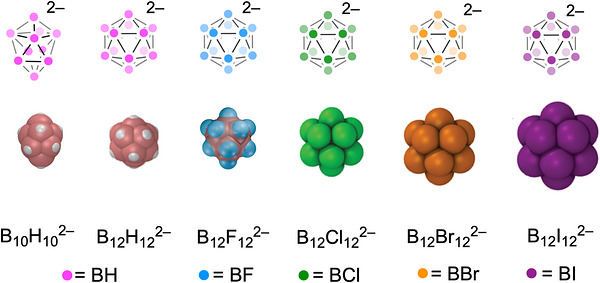
Polyhedral structures (top), molecular models (middle), and chemical formulas (bottom) of the investigated *closo*‐borate cluster anions.

Hofmeister observed that certain ions, later also referred to as chaotropic ions, increase protein solubility in water [1, 2, 3]. The specific ion effects associated with this lyotropic or Hofmeister series have led to key implementations in numerous fields ranging from nanoscience [4] to biotechnology [5, 6, 7], with notable applications in hydrogel engineering [8, 9], battery design [10, 11], biochannels [12, 13], vesicle opening [14], and cellular exocytosis [15]. Recently, larger inorganic anions, prominently of the borate cluster type (see Figure 1) but also polyoxometalates, have been found to act as superchaotropic ions, as their salting‐in effects far exceed those of conventional chaotropic ions [16, 17, 18, 19, 20]. This finding has led to a new generation of applications in biochemistry [21, 22, 23, 24, 25], cell biology [26, 27, 28, 29, 30, 31], and polymer chemistry [32, 33, 34]. In these studies, superchaotropic ions have proven useful, for example, as transmembrane carriers, but the “most superchaotropic” ions display membrane‐lytic toxic effects [27, 28, 29, 30, 31]. Consequently, there is a growing interest in fine‐tuning superchaotropic properties [35, 36, 37], especially because large cluster ions, in contrast to simple inorganic chaotropic ions, allow a wealth of covalent chemical modifications [38, 39, 40, 41, 42, 43]. This calls for measurement and predictive scaling of their chaotropic properties to allow the design of new superchaotropic ions and their rational use for biological and materials‐science oriented applications.

**FIGURE 2:**
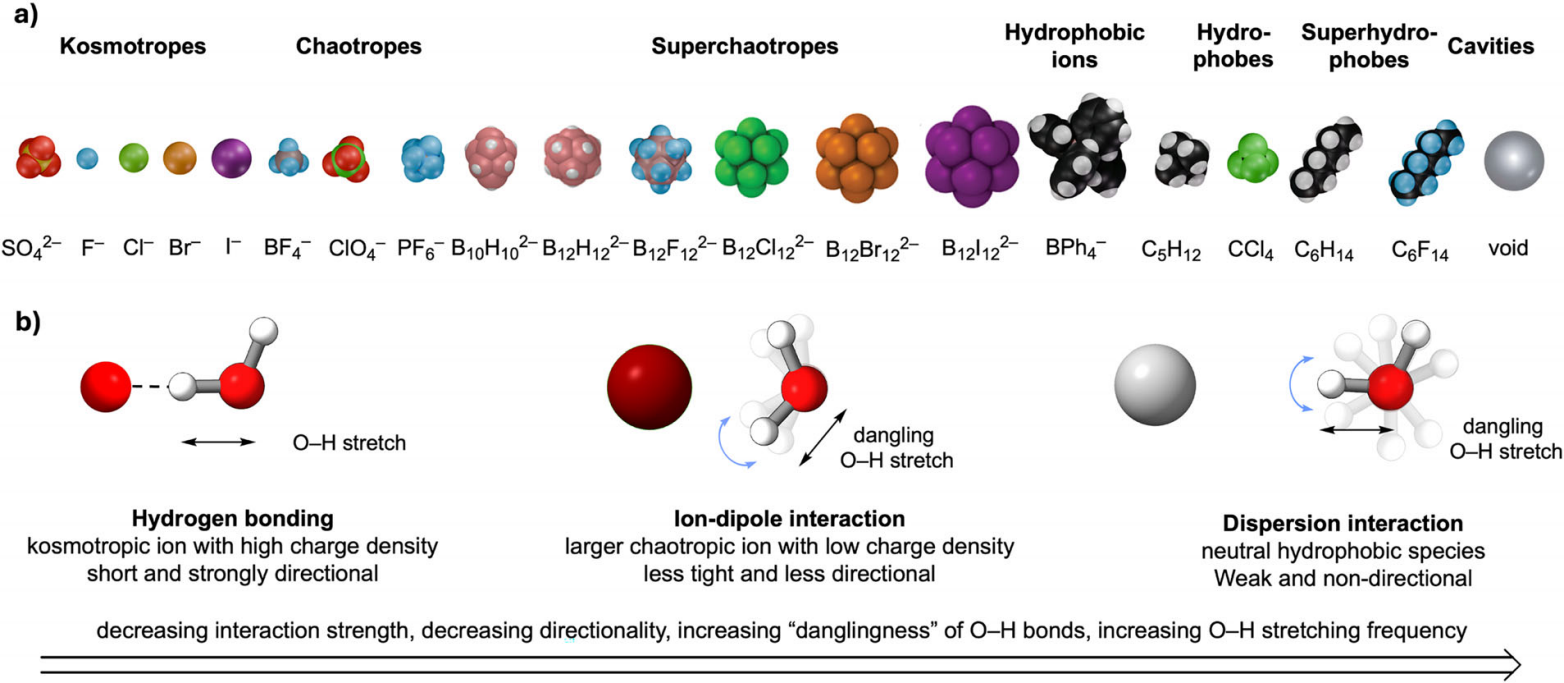
(a) Continuous scale for aqueous solvation depicting the Hofmeister series (kosmotropes and chaotropes), the expanded Hofmeister series (including superchaotropic anions), and its continuous extension (including hydrophobic ions, hydrophobes, and superhydrophobes) up to the extreme of an empty cavity as solute. C_5_H_12_: neopentane; C_6_H_14_: *n*‐hexane, C_6_F_14_: *n*‐perfluorohexane. The classifications on top are not strict in the transition regions, for example, between chaotropes, superchaotropes, and hydrophobic ions. (b) Schematic interaction of a single water molecule solvating a kosmotrope (left), a chaotrope (middle), and a hydrophobe (right) with anticipated consequences for the strength and directionality of the interaction with the water molecule and its O─H stretching frequency.

Unfortunately, whenever salting‐in effects and other intermolecular interactions between (super)chaotropic ions and a second organic solute—either small organic molecules [16, 44], peptides [27, 28, 31, 35], proteins [20], lipids [19], macrocyclic hosts [16, 45, 46, 47], or polymers [32, 33, 34]—are investigated in water, these do not only depend on the ion itself [48] but also on the interacting partners, which modulate ion‐solute interactions and contribute their own dehydration effects. The resulting conglomerate effect makes it difficult to isolate the elementary ion‐specific contributions. This limitation, also referred to as the “context” dependence of chaotropic interactions [3, 49, 50, 51], has prevented, until today, the establishment of a robust experimental “chaotropicity scale”. While several empirical relationships have been observed based on affinity data [16, 34, 35, 45, 46, 47], cloud‐point measurements [17, 18, 47], changes in viscosity *B* coefficient [34], molecular dynamics (MD) simulations [19, 52], and calculated water‐structural enthalpies of ions [53, 54, 55], a spectroscopic scale for hydration effects of different solutes has remained elusive. Ideally, such a scale should not only cover superchaotropic and hydrophobic ions but also neutral hydrophobic and superhydrophobic solutes, which can be placed on a continuous scale for aqueous solvation, see Figure 2a; this scale extends on the left side to kosmotropic anions and on the right side to an empty cavity (vacuum) [56, 57]. Moreover, and by considering that the influence of chaotropic ions is occasionally associated with a “water‐structure breaking” effect [3, 49, 50, 51, 58], a spectroscopic inspection of the hydration shell around chaotropic and superchaotropic ions in solution seems highly pertinent, complementing studies on discrete ion‐water complexes in the gas phase [59, 60]. Accordingly, we have now measured the hydration‐shell spectra of conventional chaotropic as well as superchaotropic cluster ions and analyzed how the experimental O─H vibrational frequencies align with the aqueous solvation scale in Figure 2a, anticipating that the diverse types of solutes (kosmotropic, chaotropic, superchaotropic, hydrophobic, superhydrophobic, or an empty cavity) should show different and ideally distinct as well as predictably differing vibrational frequencies.

We have selected Raman multivariate curve resolution (Raman‐MCR) spectroscopy, which has previously proven capable of elucidating the characteristic hydration shells of both neutral and charged solutes [61, 62], along with recent studies on correlated vibrational spectroscopy [58]. The hydration‐shell spectra of different solutes show, in particular, a high‐frequency band, which has been assigned, for hydrophobic solutes, to dangling O─H bonds that reflect the weak and non‐directional interactions within their hydration shell. We followed the idea that the O─H vibrational frequencies should undergo a systematic shift from the (known) strong hydrogen bonding of kosmotropes on the left‐hand side of the solvation scale to weak (dangling) interactions for hydrophobes on the right (Figure 2b). Our present study of conventional chaotropic anions and superchaotropic borate clusters (Figure 1) fills the gap between kosmotropic and hydrophobic solutes, precisely to create a spectroscopic scale. Indeed, we show that the general trends of the vibrational frequencies of the dangling O─H bonds in the hydration‐shell of solutes are in overall agreement with the proposed continuous solvation scale and allow an approximate ordering of prototypal kosmotropes, chaotropes, hydrophobes, superhydrophobes, and even vacuum (gas). However, the superchaotropic dianions present outliers as the dangling O─H bond frequencies in their hydration shells do not fall above those of the classical chaotropes and, for the perhalogenated boron clusters, show opposing trends to the established chaotropicity order of these dianions. Gas‐phase calculations of the O─H stretching frequencies in anion—H_2_O complexes can rationalize several of the deviations for the borate cluster anions and point to the importance of the higher net negative charge of the superchaotropic anions.

## 2 Results and Discussion

Vibrational spectroscopy, and especially Raman‐MCR, is capable of elucidating hydration patterns of both neutral and charged solutes in aqueous solution [61, 62, 63]. At ambient temperature, pure water shows an O─H stretching vibrational band at 3100−3200 cm^−1^ that is characteristic of a tetrahedrally coordinated (ice‐type) structure and a second component near 3450−3460 cm^−1^ that is ascribed to a more disordered hydrogen‐bonded structure [64]. An increase in temperature results in concomitant cleavage of hydrogen bonds and a “melting away” of the 3100 cm^−1^ band, such that the higher‐frequency band eventually predominates [65]. The temperature‐dependent hydration‐shell vibrational spectra of neutral hydrophobic solutes, such as methane and neopentane (or neopentanol, to achieve sufficient solubility), can be interpreted as a composite of water molecules with tetrahedral hydrogen‐bonded structures (near 3200 cm^−1^) and also a less ordered/tetrahedral peak at ca. 3660 cm^−1^; the latter has been associated with “dangling” O─H bonds that are facing toward the hydrophobic solute [65, 66, 67]. Higher temperatures favor again the disordered over the ordered component related to hydrophobic solvation [66, 67].

While the low frequencies related to tetrahedrally structured water in pure liquid water and for water in the vicinity of hydrophobes are comparable and imply similar ice‐type ordering, the dangling O─H stretching frequencies are higher than that in pure water, which indicates weaker intermolecular (hydrogen‐bond donating) interactions of the hydrating water molecules with the solute than that between hydrogen bonded water molecules. Thus, the vibrational spectral bands in the high frequency region (those above 3450 cm^−1^) are indicative of more disordered hydration shells containing dangling O─H bonds, while strong components around 3200 cm^−1^ signal the formation of a large number of highly ordered, tetrahedrally coordinated (fully hydrogen‐bonded) water molecules in the hydration shell or direct hydrogen bonds to the solute (Figure 2b).

The right‐hand side of the solvation scale (Figure 2), with weakly interacting solutes such as hydrophobes and even empty space, was scrutinized first, which corresponds to the bottom entries in Table 1. Vibrational spectroscopy of water molecules at an alkane‐water interface yielded a vibrational O─H frequency at 3674 cm^−1^ [68], suggesting weak solute‐water interactions similar to those of neopentanol [66]. This frequency decreases (3669 cm^−1^) when the alkane‐water is exchanged by a CCl_4_‐water interface [68], because the O─H bonds coordinate more strongly (are more ordered, or less dangling) with the more polarizable perchlorinated hydrocarbon. In contrast, when a less polarizable interface is selected (a perfluorinated one) [69], the frequency increases, to 3696 cm^−1^ [68, 70, 71]. Ultimately, when no intermolecular interaction partner is offered to the dangling O─H bonds at all, namely at the air‐water interface, the frequency increases strongly, to 3705 cm^−1^ [68], which defines the situation of condensed interfacial water molecules, only slightly surpassed by an isolated gas‐phase water molecule (3707 cm^−1^, for HDO to avoid coupling) [72, 73, 74]. Accordingly, the experimental O─H dangling‐bond frequencies measured for solvation of hydrophobes (alkanes) and superhydrophobes (perfluoroalkanes) as well as an empty cavity/vacuum align very well with their positioning on the continuous scale in Figure 2.

**TABLE 1:** The O─H stretch frequency assigned to hydration‐shell water molecules (ν_solute_ …_H–OH_, in cm^−1^) around representative solutes, including conventional chaotropic and *closo*‐borate cluster anions (as alkali salts)^a^ with reference to the symmetric O—H stretching vibrations in water in different phases.

Solutes/Phases	ν_solute_ …_H–OH_	References
H_2_O solid/ice	3100	[64]
PO_4_^3−^	3300	[75]
SO_4_^2−^	3441	[75]
H_2_O liquid	3450	[76]
F^−^	3458	[77]
Cl^−^	3450	[77]
Br^−^	3463	[77]
I^−^	3480	[77]
ClO_4_^−^	3584	This work
BF_4_^−^	3610	This work
PF_6_^−^	3637	This work
B_10_H_10_^2−^ ^b^	3553	This work
B_12_H_12_^2−^	3574	This work
B_12_F_12_^2−^	3635	This work
B_12_Cl_12_^2−^	3617	This work
B_12_Br_12_^2−^	3606	This work
B_12_I_12_^2−^	3591	This work
Neopentane^c^	3661	[66]
CCl_4_	3669	[68]
CH_4_	3660	[65]
C_6_H_14_	3674	[68]
C_6_F_14_	3694	[68]
Cavity/Air	3705	[68]
H_2_O vapor^d^	3707	[72, 73, 74]

^a^ Error ±2 cm^−1^.

^b^ As ammonium salt.

^c^ Neopentanol was measured, for solubility reasons.

^d^ In HDO, deuterated to avoid coupling.

**FIGURE 3:**
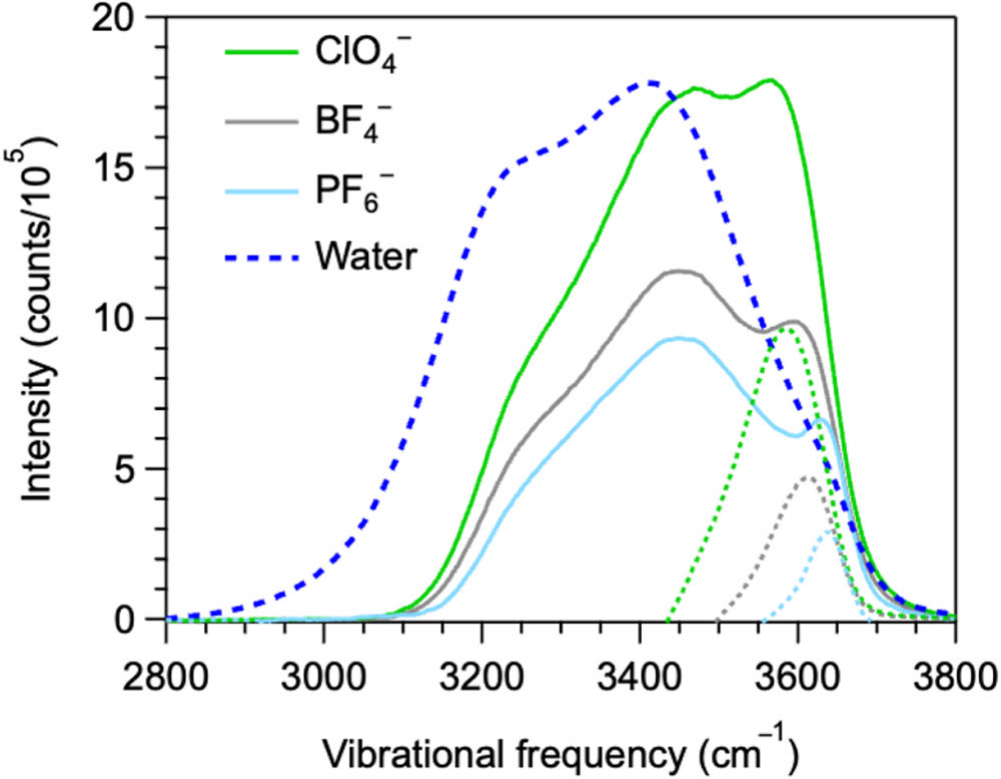
Raman hydration‐shell spectra of the chaotropic anions ClO_4_^−^ (∼0.6 M), BF_4_^−^ (∼0.6 M) and PF_6_^−^ (∼0.5 M), all as sodium salts; the Raman spectrum of pure water is shown as dashed line. The dotted peaks are obtained after subtracting the broad lower frequency bands to isolate the high frequency peaks that are assigned to dangling O─H bonds around these anions (Table 1).

Moving to the left‐hand side of the scale, previously reported hydration‐shell spectroscopic data of anionic solutes also expose characteristic vibrational frequency trends. Small or highly charged anions, which are known to be strongly solvated hydrogen bond acceptors, such as F^−^, Cl^−^, SO_4_^2−^, and PO_4_^3−^, define the kosmotropic side of the solvation scale in Figure 2a, with reported O─H stretching frequencies [57, 75, 78] comparable or falling even below (< 3460 cm^−1^) the vibrational frequency of (hydrogen‐bonded) liquid water. The O─H bonds with these anions are coordinative in nature, corresponding to actual hydrogen bonds to adjacent water molecules (“not dangling”), in some cases even stronger than those between two water molecules. However, this region of the kosmotropic anions defines only the starting point of a scale where any deviations toward higher vibrational frequencies reflect weaker hydrogen‐bonding and higher dangling O─H character around increasingly chaotropic anions. Indeed, when examining the Raman hydration‐shell spectra, the frequencies of the dangling O─H in the hydration shell of larger halide ions, namely of Br^−^ (3463 cm^−1^) and I^−^ (3480 cm^−1^), increase gradually [77, 78], indicative of weaker hydrogen bonding to these anions or more “dangling character” of O─H bonds pointing toward the solute. Our measurements of the Raman hydration‐shell spectra of the classical chaotropic anions, going beyond the heaviest halide (I^−^), namely the larger globular singly charged anions ClO_4_^−^, BF_4_^−^, and PF_6_^−^, show that this systematic trend toward higher frequencies continues, reaching values of 3584, 3610, and 3637 cm^−1^, see Table 1 and Figure 3. These higher frequencies, which fall nevertheless below those of hydrophobic solutes, can be attributed to less strongly hydrogen‐bonded and increasingly dangling O─H bonds and reflect weaker electrostatic interactions with the larger anions, attributable to decreasing partial atomic charges and atomic or molecular charge densities. This region between 3460 and 3640 cm^−1^ for the larger, singly charged anions defines the chaotropic region of the continuous hydration scale in Figure 2. With a little hindsight, Lindgren and coworkers have previously found the same trend for this set of inorganic ions by measuring O─D stretching frequencies by IR spectroscopy in HDO‐doped aqueous solutions [57, 62, 79].

**FIGURE 4:**
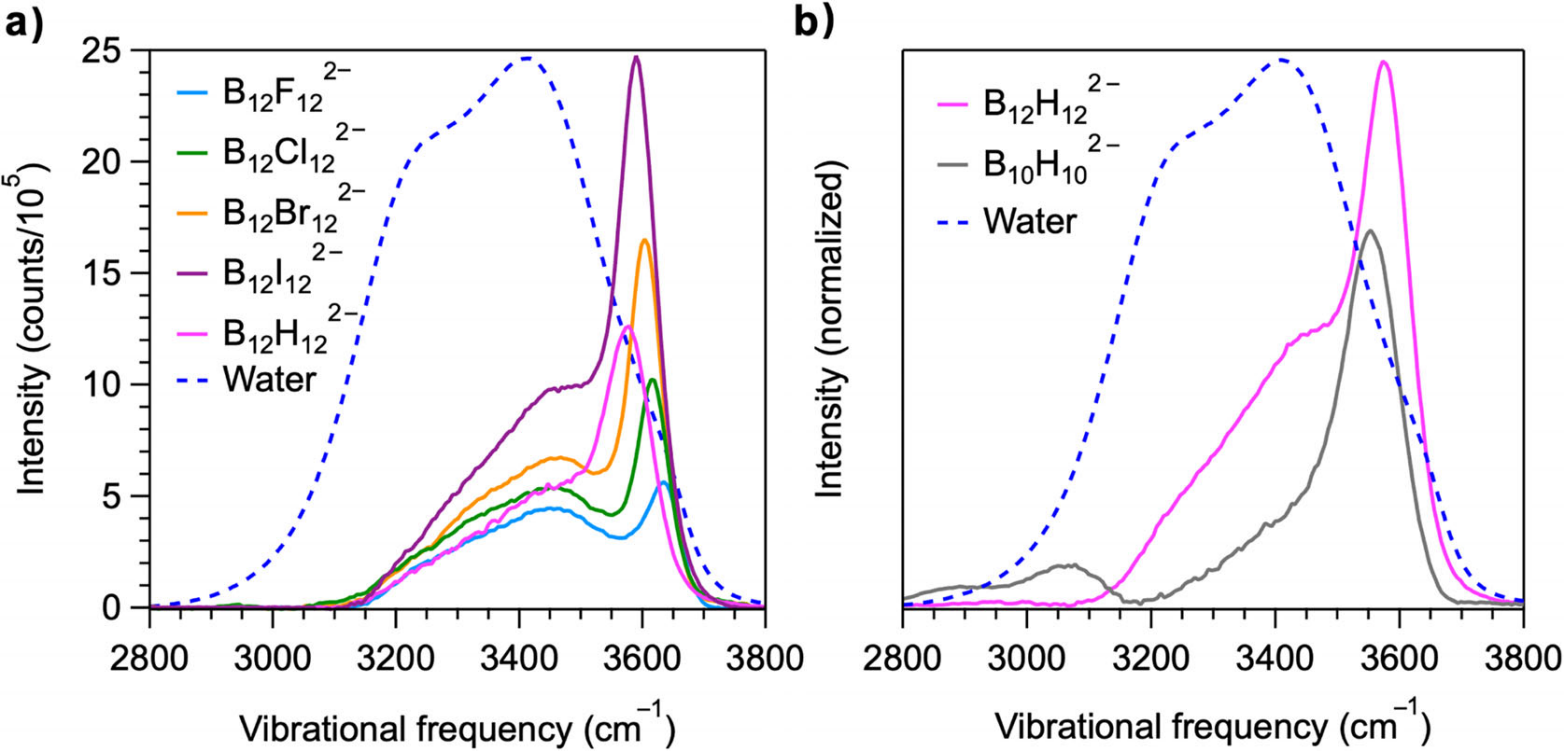
Raman hydration‐shell spectra of superchaotropic anions, (a) of dodecaborates B_12_X_12_^2−^ (as alkali salts) and (b) of (protiated) decaborate B_10_H_10_^2−^ and dodecaborate B_12_H_12_^2−^ anions (as ammonium and sodium salt, respectively); the Raman spectrum of pure water is shown as dashed line and scaled for reference. Note that the feature between 2800 and 3100 cm^−1^ in (b) stems from the interaction of water with the NH_4_^+^ counter cation of B_10_H_10_^2−^.

We found that the vibrational frequencies of the dangling O─H bonds around solutes are characteristic of the chaotropicity of anions. On one hand, the chaotropicity trend for the conventionally employed monoanions, namely Br^−^ < I^−^ < ClO_4_^−^, BF_4_^−^ < PF_6_^−^, was well reproduced by the relative dangling O—H frequencies. On the other hand, the frequency range of these chaotropic anions (3460–3640 cm^−1^) fell distinctly below the range for the hydrophobes (3660 ± 15 cm^−1^), in line with the solvation scale (Figure 2). It was therefore of interest to spectroscopically investigate the hydration shells of superchaotropic anions. In this study, we focused on the highly stable *closo*‐borate cluster anions, because they do not undergo speciation or hydrolysis as most other superchaotropic polyoxometalates do [80]. Accordingly, we recorded the Raman spectra and the background‐subtracted Raman hydration‐shell spectra for the sodium salts of the dodecaborate series, B_12_X_12_^2−^ (with X = H, F, Cl, Br, and I), see Figure 4a (see Figure S1 for uncorrected and background‐subtracted Raman spectra over the entire wavelength range). Indeed, all spectra displayed a high‐frequency band that, leaning on previous measurements with hydrophobic solutes [61, 65, 66, 67], can be assigned to water “dangling”  O—H groups that surround and interact with the dodecaborate anions (note that the alkali counter cations do not produce comparable signals in this this type of spectroscopy) [61, 77]. However, against expectation, the frequencies of these “dangling O—H bonds” fell more in the range of the strong conventional chaotropes (ClO_4_^−^, BF_4_^−^, and PF_6_^−^) but did not exceed them. Moreover, the frequencies did not systematically increase with ion size and lower charge density (all ions are doubly negatively charged), but in the order B_12_H_12_^2−^ < B_12_I_12_^2−^ < B_12_Br_12_^2−^ < B_12_Cl_12_^2−^ < B_12_F_12_^2−^, see Table 1. Therefore, on the dangling O─H bond frequency scale, the superchaotropic ions behave more as outliers.

The exceptional behavior of the parent compound, namely that the frequency of B_12_H_12_^2−^ falls below those of the halogenated ones (B_12_X_12_^2−^, X = F, Cl, Br, and I) can be rationalized best, because boron hydrides are known to form a special intermolecular interaction with water, namely dihydrogen bonds of the type B─H^δ−^··· ^δ+^H─O [81, 82, 83, 84]. This is due to the peculiar polarization of the B─H bond, that is opposite to C─H bonds and results in a considerable negative partial charge of −0.25 on the hydrogen atoms in B_12_H_12_^2−^ [81, 85]. This additional interaction accounts for the lower water O─H stretching frequencies of the protiated boron clusters, which has also been calculated for the monohydrated anion in the gas phase [83]. The same interaction should apply for the smaller, also doubly negatively charged B_10_H_10_^2−^ cluster [35, 82]. We tested this hypothesis experimentally and, indeed, this anion showed an even lower dangling O─H frequency (Figure 4b). It should be noted that irregular trends of the dangling O─H frequencies due to specific intermolecular interactions have been observed previously, for example, tetraphenylborate (BPh_4_^−^) and benzyl alcohol, two solutes otherwise recognized as being hydrophobic ions or containing hydrophobic residues (phenyl), also show dangling O─H frequencies which are lower than expected due to a special type of intermolecular water‐anion interactions, O─H···π bonding in this case [66, 86, 87]. In contrast, hydrophobic ions devoid of aromatic groups, for example, carboxylic acids and also tetraalkylammonium cations, display the expected dangling O─H frequencies (near 3660 cm^−1^) characteristic of hydrophobic hydration, along with the temperature‐dependent “ice‐type” band near 3200 cm^−1^ [76].

The ordering for the halogenated superchaotropic clusters is opposite to expectation, because their chaotropicity increases with ion size and lower charge density, while the dangling O─H frequencies show the opposite trend. Electrostatic effects related to partial atomic charges of the halogens cannot account for this trend either, because they decrease (in absolute terms) from F (−0.47) to Cl (−0.15) to Br (−0.10), to I (+0.04) [88]. Larger dispersion interactions for the heavier halogenated clusters [35, 89] could potentially increase the strength of the dangling O─H···X bonds, but gas‐phase experiments (negative ion photoelectron spectroscopy) have actually shown weaker boron cluster‐water interactions for the iodinated one (B_12_I_12_^2−^) than for the fluorinated one (B_12_F_12_^2−^), both in the monohydrated as well as polyhydrated complexes, B_12_X_12_^2−^•(H_2_O)*_n_*, *n* = 1–6 [84]. Moreover, the calculated water O─H stretching frequencies in the iodinated monohydrated complex are higher than those in the fluorinated counterpart, opposite to what is observed in the liquid‐water Raman hydration‐shell spectra, where, evidently, the brominated and iodinated clusters appear to interact somewhat more strongly with the O─H bonds than the chlorinated and fluorinated clusters do. In short, the trend of the dangling O─H bond frequencies for the halogenated clusters is inconsistent with expectations based on their chaotropicity [19, 20, 35], charge density considerations, and the continuous scale for aqueous hydration shown in Figure 2.

**FIGURE 5:**
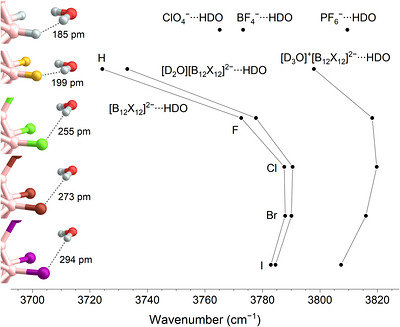
Calculated “dangling” O–H stretching frequencies in mono‐ and dihydrated complexes of chaotropic monoanions (top) and superchaotropic dodecaborate dianions (connected lines). On the left, the optimized structures of the [B_12_X_12_]^2−^ ··· H–OD complexes are depicted; note that the calculated frequencies are not scaled and only relative trends with the experimental frequencies (Table 1) should be compared.

To assess whether simple models could facilitate the understanding of the unexpected trend observed in the series of halogenated [B_12_X_12_]^2−^ ions, the binding of a single water molecule to dodecaborate dianions and the chaotropic anions ClO_4_^−^, BF_4_^−^ and PF_6_^−^ was investigated using DFT calculations. All energy optimizations yielded geometries expected from charge‐dipole interactions (Figure 2b), with both hydrogens of HDO oriented in a bifurcated manner toward the anions. However, the differences in interactions dependent on the dodecaborate substituent X become apparent (Figure 5, left): while small halogens exhibit strongly directional H···X interactions, the σ‐hole of the larger halogens directs the H^δ+^ of water toward the negatively polarized B─X bonds [88, 90].

HDO was selected to decouple the two OH‐stretching vibrations, thereby enabling the determination of the dangling OH frequency—a method also widely applied in experimental studies [72, 73, 74]. The calculated dangling OH frequencies for various chaotropic and superchaotropic anions are shown in Figure 5. First, it is gratifying to see that this isolated model system (one water molecule attached to one ion) reproduces the experimental observation that the dangling OH frequencies of dodecaborate dianions in aqueous solution fall in the same frequency range as those of the chaotropic monoanions and do not exceed them. Nevertheless, the experimental trend of increasing dangling OH frequency from X = I to X = F is not reproduced in this isolated model system. Specifically, the dangling OH frequencies in [B_12_F_12_]^2−^···HDO and [B_12_H_12_]^2−^···HDO exhibit strong red shifts compared to the other clusters (Figure 5, middle), different from the solution phase measurements.

We hypothesized that the “vibrational Stark effect”, known to induce red shifts in the OH bands of water exposed to electric fields, accounts for the observed discrepancy. This effect would most significantly affect the OH bonds of water molecules interacting with small, highly charged ions, particularly [B_12_H_12_]^2−^ and [B_12_F_12_]^2−^, where a substantial portion of the negative charge resides on the outer sphere of the ions. In solution, this effect would be partially compensated by charge screening due to solvation and the overall charge‐balanced environment. Such screening may explain the different trend between the isolated model system and condensed‐phase experimental data.

To simulate the influence of solvation, an additional water molecule was attached to the opposite side of this isolated model system, ensuring no direct interaction with the DOH probe molecule. This “computational experiment” demonstrates a shift in the vibrational frequency toward higher wavenumbers, with the effect being more pronounced for smaller ions. To model a reduced effective charge, which is present in a charge‐balanced solution, an [H_3_O]^+^ was added to the opposite side of the cluster. The reduced charge induces a blueshift in all dangling OH frequencies, consistent with a diminished vibrational Stark effect. Notably, the magnitude of this effect varies significantly depending on the dodecaborate substituent X, resulting in a revised trend across the halogen series that closely resembles the experimental trend (Figure 5).

While the strong interaction of a single water molecule with [B_12_H_12_]^2−^ and [B_12_F_12_]^2−^ is dominated by electrostatic effects, the direct ion‐dipole interaction and the electric field experienced by the dangling OH bond are significantly weaker for the larger [B_12_I_12_]^2−^ ion. Instead, for large X, the high polarizability of the dianion [35] contributes substantially to the interaction with water through induction and dispersion interactions, which are less sensitive to charge screening by solvation and countercharges. Although no quantitative comparison to experimental solution‐phase data (Figures 3 and 5) can be drawn from the simple isolated model, the series highlights the importance of charge state, solvation, and effective charge in determining the dangling OH frequency. The similar dangling OH frequencies observed for superchaotropic dodecaborate dianions and standard chaotropic anions can accordingly be rationalized by considering their distinct higher net charge in combination with charge‐screening effects, which determine the trend along the series of halogen substituents.

Besides the dangling O─H bond frequencies, the band intensities in the borate cluster series show a meaningful trend with the number of water molecules in the corresponding solvation shell [57, 77]. As expected, the hydration‐shell OH band intensities in Figure 5a increase with the size of the cluster and halogen type, that is, B_12_I_12_^2−^ > B_12_Br_12_^2−^ > B_12_Cl_12_^2−^ > B_12_F_12_^2−^. However, the hydrogenated dodecaborate, B_12_H_12_^2−^, falls out of this simple correlation, because it is the smallest cluster, but its dangling O─H band has an intermediate intensity, which is likely due to the special type of dihydrogen bonding (see above). When comparing the Raman dangling O─H bond intensities of the two protiated boron clusters, B_10_H_10_^2−^ and B_12_H_12_^2−^, shown in Figure 5b, the former has again a lower intensity, as expected given its smaller size (Table 1).

In the context of the band intensities, it is important to note that the frequencies of the dangling O–H bonds reflect a molecular spectroscopic property of individual water molecules directly adjacent to the anions. However, most effects related to the chaotropicity of anions are related to collective/ensemble effects of their entire hydration shells. This means that any scale based on dangling O–H frequencies in the hydration shells of the anions does not necessarily have to coincide with their macroscopic chaotropic effects, but that the aggregate effects on the entire hydration shell may be more diagnostic. To the degree that the *offset* of the O–H water stretching band envelope (the integrated intensity) toward higher frequencies may be a better measure of the chaotropicity of the anions one would indeed expect a larger shift of the band intensity for the more chaotropic anions. This is experimentally observed, that is, the O–H vibrational band for the more chaotropic iodinated and brominated borate clusters is visibly more strongly displaced toward higher frequencies around 3600 cm^−1^ than for the smaller borate clusters (Figure S1a), owing to their larger hydration shell/surface area. Consequently, an anion may behave phenomenologically as a stronger chaotrope even if the dangling O–H water frequencies of its surrounding individual water molecules may be indicative of a stronger cluster‐water interaction (or lower O─H bond frequencies) compared to another anion. This may account for the contrasting trend in the halogenated dodecaborate cluster series.

**FIGURE 6:**
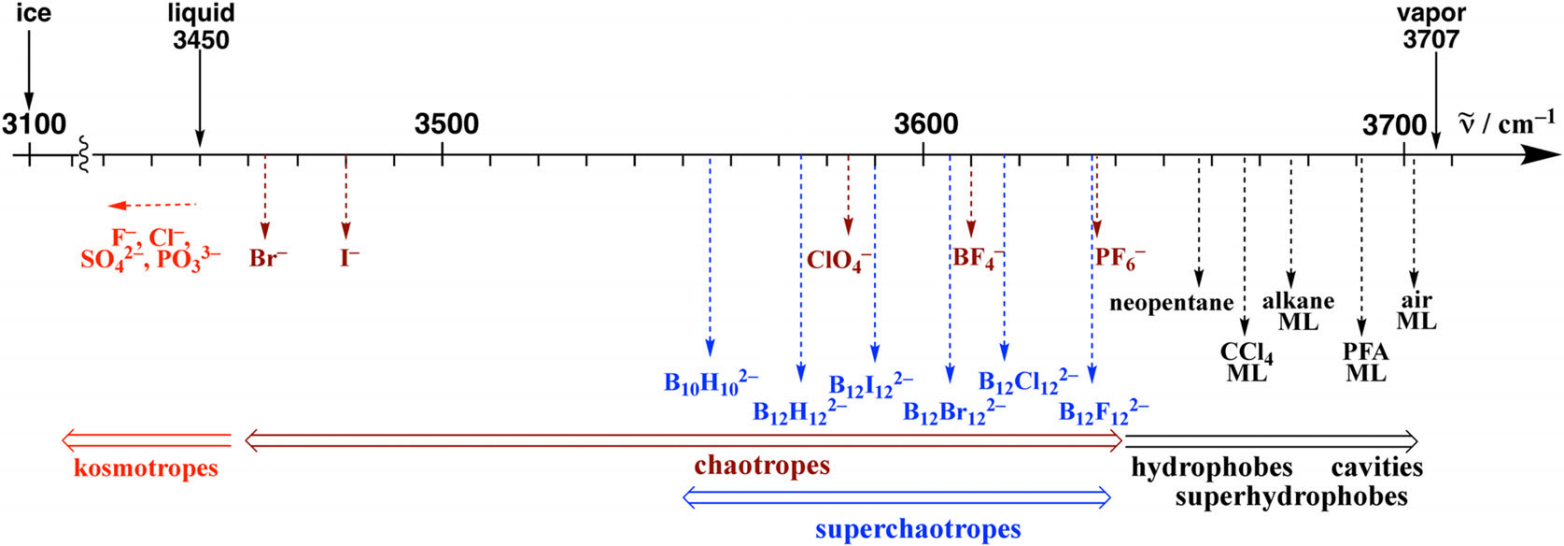
Raman‐spectroscopic scale for dangling O‐H frequencies of different solutes. The scale features “globular” ions and excludes dipolar and aromatic solutes (due to additional dipole–dipole and O–H—π interactions).

The combined results for the Raman‐spectroscopic scale of dangling O─H bond frequencies for all discussed solutes are shown in Figure 6. We focused on nonpolar and nonaromatic solutes, and on “globular” ions, as solutes with dipole moments and aryl rings lead to additional interactions. Kosmotropic ions are clearly grouped on the left side, hydrophobic and superhydrophobic species and monolayers, as well as the gas phase/air are located on the right side. Chaotropic ions and superchaotropic ions are grouped in the middle of the spectroscopic scale, but there is substantial overlap, for reasons outlined above. Therefore, the dangling O─H scale is roughly in agreement with the continuous solvation scale, with superchaotropic ions not strictly adhering to the trend. This is consistent with the assumption that the strength of intermolecular interactions decreases upon moving from left to right, from strong hydrogen bonding for kosmotropic anions, to weaker ion‐dipole interactions that decrease with the size of the chaotropic and superchaotropic anions, to even weaker dispersion interactions that decrease with the (bulk) polarizability [69] of the interacting species until there is no interaction at all, with the gas phase or an empty cavity, see also Figure 2b. Different parameters, including direct ion–water interactions and charge screening effects act in concert and may lead to apparent outliers on the spectroscopic scale, for example, for the halogenated borate cluster series.

To the degree that the O─H frequencies report on the water structure, with fully tetrahedrally hydrogen‐bonded ice‐type water on the left and fully unstructured (gas‐phase) water on the right, ions positioned on the left side reinforce the tetrahedral water structure, while other solutes decrease water structure to various extent. However, other changes from the hydration‐shell spectra also deserve attention. In particular, the dissolution of hydrophobic species not only leads to dangling O─H bonds showing high frequencies, but also reinforces water structure, as evidenced by an additional band at 3200 cm^−1^ which becomes prominent at ambient and lower temperature [65]. Therefore, hydrophobes show a compensating water‐structuring of water molecules whose O─H bonds are not directly pointing to the solute, reminiscent of the original iceberg model of hydrophobic hydration [65, 91]. This low‐frequency band and “structural compensation” is not evident from the hydration‐shell spectra of the chaotropic and superchaotropic species (see Figure 3 and Figure 4a). Rather, their hydration strengthens the 3450 cm^−1^ band of water, apparent through a separate band or shoulder in their hydration‐shell spectra, meaning that effectively some of the tetrahedral water molecules in the hydration shell are converted into water molecules with only three or two hydrogen bonds [63], corresponding to a net water‐structure breaking effect. Accordingly, the hydration‐shell spectra allow a differentiation between chaotropic and superchaotropic anions (hydration‐shell band near 3450 cm^−1^ and a high‐frequency band below 3640 cm^−1^, this work) and hydrophobes (high‐frequency band above 3645 cm^−1^ and low‐frequency contribution around 3200 cm^−1^, especially at low temperature, see refs [65, 66, 67]), while kosmotropic ions lack the high‐frequency band. This may serve as a spectroscopic fingerprint. The spectroscopic difference corroborates the notion that superchaotropes do not behave as hydrophobic species, but that they display a hydration pattern and associated properties on their own right. Indeed, the chaotropic effect differs from the hydrophobic effect also thermodynamically because it leads to enthalpy‐driven association phenomena rather than entropy‐driven ones [34, 56].

## 3 Conclusions

In summary, the Raman hydration‐shell spectra of prototypal chaotropic and superchaotropic anions have been measured for the first time. The combination of these experimental data with reported vibrational spectroscopic data for the hydration of kosmotropic, hydrophobic, and superhydrophobic solutes lends general support for the idea that the O─H stretching frequencies of hydration‐shell water molecules (“dangling O─H”) can be used to construct an approximate continuous scale for aqueous solvation [16, 57]. For example, the O─H stretching frequencies of common chaotropes lie above those of common kosmotropes, while those of superchaotropic halogenated dodecaborates (B_12_X_12_^2−^) overlap or exceed those of the classical chaotropes, and for some derivatives come close to those of the dangling O─H bonds otherwise only observed for hydrophobic solutes. DFT calculations for monohydrated anion complexes account for this unexpected overlap between chaotropes and superchaotropes but also pinpoint a significant influence of the net ion charge state and potential charge screening effects on the vibrational frequencies of the O–H bonds in the hydration shell of the ions. They reveal that the use of a dangling O–H frequency scale as a measure for monovalent versus divalent chaotropic anions is not straightforward. Nevertheless, the spectroscopic scale displays systematic trends, and deviations can be rationalized in terms of competitive intermolecular interactions and the net charge of the anions. Moreover, a differentiation of kosmotropic anions from (super)chaotropic anions from (super)hydrophobic solutes is possible using Raman hydration‐shell spectroscopic fingerprints, at least for the investigated nonpolar solutes and globular anions.

## Author Contributions

**Werner M. Nau**: conceptualization, funding acquisition, writing – original draft, project administration, and supervision. **Denilson Mendes de Oliveira**: investigation, methodology, writing – review and editing, and formal analysis. **Andres S. Urbina**: investigation, writing – review and editing, methodology, and formal analysis. **Harald Knorke**: investigation, writing – review and editing, software, and formal analysis. **Jonas Warneke**: conceptualization, writing – review and editing, supervision, and funding acquisition. **Andrea Barba‐bon**: writing – review and editing, software, and formal analysis. **Dor Ben‐amotz**: conceptualization, funding acquisition, writing – review and editing, and supervision.

## Conflicts of Interest

The authors declare no conflicts of interest.

## Supporting information




**Supporting File 1**: The authors have cited additional references within the Supporting Information [16, 65, 92].

## Data Availability

The data that supports the findings of this study are available in the Supporting Information of this article.
