# Supplementary material for: Specific Ion Effects of Chaotropic and Superchaotropic Anions Probed by Raman Hydration‐Shell Spectroscopy
Source: Angew Chem Int Ed Engl. 2026 May 22;65(27):e6630432. doi: 10.1002/anie.6630432 (PMC13327565; doi:10.1002/anie.6630432)
Supplement: Supplementary file 1 — Supporting File 1: The authors have cited additional references within the Supporting Information [16, 65, 92]. [file ANIE-65-e6630432-s001.pdf]

Supporting Information for

Specific Ion Effects of Chaotropic and Superchaotropic Anions Probed by Raman Hydration-Shell Spectroscopy

Werner M. Nau,[a]* Denilson Mendes de Oliveira,[b] Andres S. Urbina,[b] Harald Knorke,[c] Jonas Warneke,[c]* Andrea Barba-Bon,[a] and Dor Ben-Amotz[b]

[a] Prof. Dr. Werner M. Nau, Dr. Andrea Barba-Bon
School of Science,
Constructor University
Campus Ring 1, 28759 Bremen, Germany
E-mail: wnau@constructor.university

[b] Prof. Dr. Dor Ben-Amotz, Dr. Denilson Mendes de Oliveira, Dr. Andres S. Urbina
Department of Chemistry
Purdue University
West Lafayette, Indiana 47907, USA

[c] Prof. Dr. Jonas Warneke, Dr. Harald Knorke
Wilhelm-Ostwald-Institut für Physikalische und Theoretische Chemie, Universität Leipzig, Linnéstr. 2, 04103 Leipzig, Germany, and Leibniz Institute of Surface Engineering (IOM), Permoserstr. 15, 04318, Leipzig, Germany
E-mail: jonas.warneke@uni-lepipzig.de

Experimental Details

Dodecaborate clusters including $Na_2B_{12}H_{12}$, $Na_2B_{12}Cl_{12}$, $Na_2B_{12}Br_{12}$, and $Na_2B_{12}I_{12}$ were taken from a previous study;[1] $K_2B_{12}F_{12}$, $NaBF_4$, and $NaClO_4$ were from Sigma-Aldrich (Darmstadt, Germany) and $NaPF_6$ from Alfa Aesar (Massachusetts, USA).

Emission spectra with excitation at λ_{ex} = 515 nm were measured with a Jasco FP-8500 as well as a Varian Eclipse spectrofluorometer in a 3.5 ml quartz cuvette from Hellma Analytics (Müllheim, Germany) in Millipore water at 1 mM salt concentration. The samples did not afford any detectable emission signal beyond the characteristic Raman scattering band at ca. 624 nm, allowing further investigation by Raman spectroscopy.

Raman spectra were obtained at 20 °C using an Ar-ion 514.5 nm laser with ~20 mW of power at the sample, with the previously described instrumental set-up (Figure S1a).[2] Aqueous solutions of the boron cluster sodium salts $Na_2B_{12}X_{12}$ (X = H, Cl, Br, I), $K_2B_{12}F_{12}$, $(NH_4)_2B_{10}X_{10}$ (all 100 mM), $NaBF_4$ (600 mM), $NaClO_4$ (600 mM), and $NaPF_6$ (500 mM) were prepared and transferred to glass capillaries. It should be noted that some additional boron cluster samples ($Cs_2B_{10}Cl_{10}$, $Cs_2B_{10}Br_{10}$, $Cs_2B_{10}I_{10}$, $KB_{21}H_{18}$,) were insufficiently soluble (< 5 mM) to afford sufficiently strong signals and were therefore excluded from further study. For the sufficiently soluble salts, a long-working distance 20× objective (Mitutoyo Inc.) was used to focus the laser at the center of the capillary and collect the backscattered Raman light. Duplicate 5-min spectra were acquired for all samples to produce high signal-to-noise Raman spectra at the water OH band. Self-modeling curve resolution (SCMR) was used to obtain solute-correlated (SC) spectra from pure water and the solution spectra. The resulting SC spectra (Figure S1b) contain spectral features arising from intramolecular vibrational modes of the solute ions as well as from solvent molecules whose vibrations are perturbed by the solute. The solvent component is constrained to the experimentally measured spectrum of pure water while the SC component is equivalent to the minimum-area (non-negative) difference between the solution and pure water spectra.

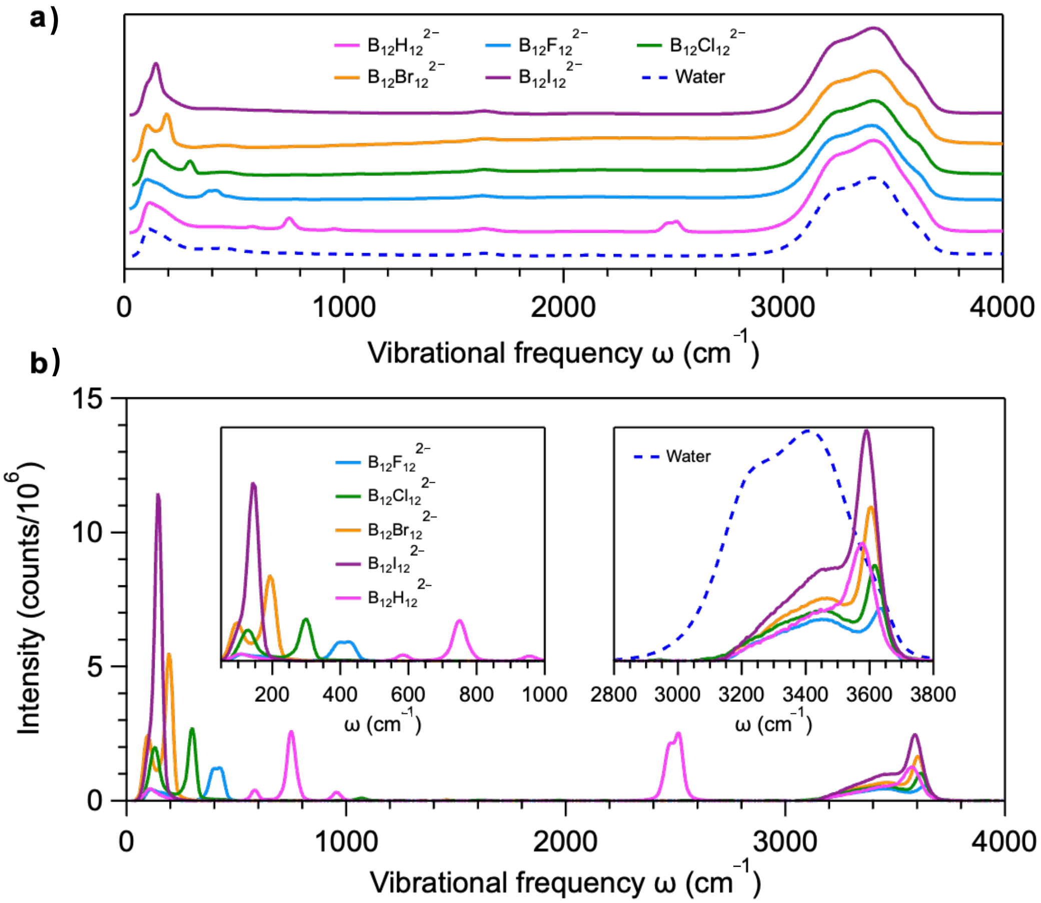

Figure S1. **a)** Full-range Raman spectra of sodium dodecaborate salts ($Na_2B_{12}X_{12}$, 100 mM) and **b)** full-range Raman-MCR solute-correlated spectra; the reference spectra of neat water are shown as blue dashed lines.

Computational Details

Water-ion interactions were investigated using Gaussian 16, Revision C.02,[3] employing the B3LYP functional with the def2-TZVPP basis set. Geometry optimizations were performed with tight convergence criteria, including empirical dispersion corrections (GD3BJ). Vibrational frequency calculations were carried out to confirm the nature of the stationary points. An ultrafine integration grid (SuperFineGrid, Acc2E=13) was used to ensure numerical accuracy. The results are depicted in Figure 5 of the manuscript.

References

[1] K. I. Assaf, M. S. Ural, F. Pan, T. Georgiev, S. Simova, K. Rissanen, D. Gabel, W. M. Nau, Water Structure Recovery in Chaotropic Anion Recognition: High-Affinity Binding of Dodecaborate Clusters to γ-Cyclodextrin. *Angew. Chem. Int. Ed.* **2015**, *54*, 6852-6856.

[2] X. Wu, W. Lu, L. M. Streacker, H. S. Ashbaugh, D. Ben-Amotz, Methane Hydration-Shell Structure and Fragility. *Angew. Chem. Int. Ed.* **2018**, *57*, 15133-15137.

[3] R. C. Gaussian 16, M. J. Frisch, G. W. Trucks, H. B. Schlegel, G. E. Scuseria, M. A. Robb, J. R. Cheeseman, G. Scalmani, V. Barone, G. A. Petersson, H. Nakatsuji, X. Li, M. Caricato, A. V. Marenich, J. Bloino, B. G. Janesko, R. Gomperts, B. Mennucci, H. P. Hratchian, J. V. Ortiz, A. F. Izmaylov, J. L. Sonnenberg, D. Williams-Young, F. Ding, F. Lipparini, F. Egidi, J. Goings, B. Peng, A. Petrone, T. Henderson, D. Ranasinghe, V. G. Zakrzewski, J. Gao, N. Rega, G. Zheng, W. Liang, M. Hada, M. Ehara, K. Toyota, R. Fukuda, J. Hasegawa, M. Ishida, T. Nakajima, Y. Honda, O. Kitao, H. Nakai, T. Vreven, K. Throssell, J. A. Montgomery, Jr., J. E. Peralta, F. Ogliaro, M. J. Bearpark, J. J. Heyd, E. N. Brothers, K. N. Kudin, V. N. Staroverov, T. A. Keith, R. Kobayashi, J. Normand, K. Raghavachari, A. P. Rendell, J. C. Burant, S. S. Iyengar, J. Tomasi, M. Cossi, J. M. Millam, M. Klene, C. Adamo, R. Cammi, J. W. Ochterski, R. L. Martin, K. Morokuma, O. Farkas, J. B. Foresman, and D. J. Fox,, Gaussian, Inc., Wallingford CT. **2016.**
